# Dynamische Übergabeschurre zur Effizienzsteigerung von Gurtbandförderern

**DOI:** 10.1007/s00501-021-01084-4

**Published:** 2021-02-05

**Authors:** Michael Prenner

**Affiliations:** Lehrstuhl für Bergbaukunde, Bergtechnik und Bergwirtschaft Arbeitsgruppe Fördertechnik und Konstruktionslehre, Montanuniversität Leoben, Franz-Josef-Straße 18, 8700 Leoben, Österreich

**Keywords:** Dynamische Übergabeschurre, Gurtbandförderer, Effizienzsteigerung, Verschleißreduktion, Energierückgewinnung, Dynamic transfer chute, Belt conveyor, Improvement in efficiency, Wear reduction, Energy recovery

## Abstract

Bei der Übergabe von Schüttgütern von einem Gurtbandförderer auf einen weiteren werden sogenannte Übergabeschurren benötigt. Diese Schurrensysteme verursachen in Abhängigkeit des zu fördernden Schüttgutes oft starken Verschleiß, Anbackungen und eine ungünstige Materialführung. Der Verschleiß tritt dabei sowohl an den Schurrenwänden als auch am nachgeschalteten Fördergurt auf. Anbackungen an den Schurrenwänden entstehen durch den oft vorkommenden kohäsiven Feinkornanteil von Schüttgütern. Speziell bei feuchtem Schüttgut kann dies zu Verstopfungen der Schurren führen. Eine optimale Materialführung zeichnet sich durch eine mittige Beladung des nachfolgenden Förderers, einer Aufgabe des Schüttgutes in Förderrichtung des nachfolgenden Gurtbandförderers und einer möglichst verschleißarmen Umlenkung des Schüttgutes in der Schurre aus. Da herkömmliche starre Schurrensysteme die an sie gestellten Aufgaben oftmals nicht optimal erfüllen können, wird derzeit am Lehrstuhl für Bergbaukunde, Bergtechnik und Bergwirtschaft – Arbeitsgruppe Fördertechnik und Konstruktionslehre in Kooperation mit den Unternehmen ScrapeTec Trading GmbH, ScrapeTec GmbH und Wanggo Gummitechnik GmbH ein dynamisches Schurrensystem entwickelt, welches die gestellten Aufgaben signifikant besser erfüllen wird.

## Abstract (en)

At transfer points for bulk materials, transfer chutes are required between two belt conveyors. Depending on the conveyed bulk material, these chute systems often cause wear, caking, and poor material handling. Wear occurs both on the chute walls and on the downstream conveyor belt. Caking on the chute walls is oftencaused by the cohesive fine-grained particles of the bulk material. Especially wet bulk material can lead to clogging of the chutes. Bulk material handling is ideal when the bulk material is loaded centrally onto the downstream conveyor. Furthermore, feeding in conveying direction of the downstream belt conveyor and a material deflection by the chute with minimum wear are also required for ideal bulk material handling. At the “Chair of Mining Engineering and Mineral Economics—Conveying Technology and Design Methods” in cooperation with the companies ScrapeTec Trading GmbH, ScrapeTec GmbH and WanggoGummitechnik GmbH, a new dynamic transfer chute is currently being developed, since standard chute systems are often unable to optimally perform the tasks assigned to them.

## Funktionsprinzip des dynamischen Schurrensystems

**Figure 1:**
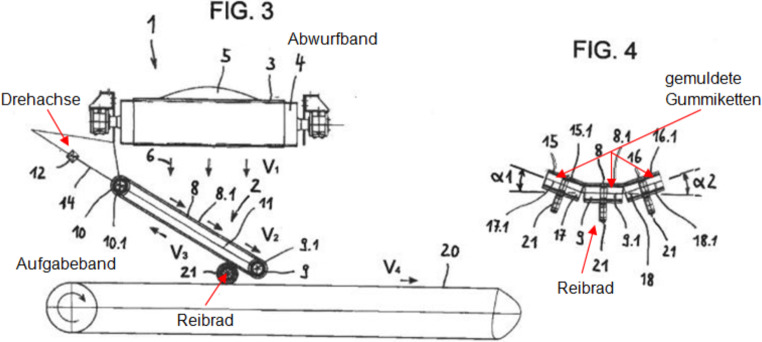

Abb. 1 zeigt das Funktionsprinzip des Systems, welches im Jahr 2019 zum Patent angemeldet wurde. Das dynamische Schurrensystem besteht aus mindestens drei zu einer Muldung zusammengeführten umlaufenden Gummiketten mit entsprechender Stützkonstruktion, wie sie z. B. für Baggerfahrwerke eingesetzt werden. Das vom Abwurfband abgegebene Schüttgut wird vom Kettensystem aufgenommen und gerichtet auf das Abgabeband aufgegeben. Die Ketten können mit oder ohne Querstollen ausgeführt werden. Die Ketten berühren sich entweder an den Längskanten oder überdecken einander, um ein Durchrieseln des Schüttgutes zwischen den einzelnen Kettensträngen zu verhindern. Die Achsen der Kettensterne sind kardanisch miteinander verbunden, um einen Gleichlauf der Ketten zu gewährleisten. Das System wird an einer Übergabestelle zwischen zwei Gurtbandförderern (Abwurfband-Aufgabeband) eingebaut, wobei es sich an zwei „Punkten“ abstützt. Der erste „Punkt“ ist eine Drehachse, welche in der Regel parallel zur Umlenktrommel des Aufgabebandes orientiert ist. Das System wird um diese Achse verdrehbar aufgehängt bzw. gelagert. Der zweite „Punkt“ ist das für die Energieübertragung verwendete Reibrad, auf dem das Untertrum der mittleren Kette aufliegt. Das Übergabesystem verfügt über keinen eigenen Antrieb. Die Energieübertragung erfolgt vom Aufgabeband auf das Reibrad und weiter auf die mittlere Kette des dynamischen Übergabesystems. Die Reibkraft wird in der Regel über die Eigenmasse des Systems erzeugt, da es in einer Ebene drehbar aufgehängt ist und sich am Reibrad abstützt. Bei Bedarf kann auch eine zusätzliche Verstelleinrichtung montiert werden, welche die Anpresskraft erhöht. Je nach Massenstrom und Übergabehöhe müssen die Ketten durch das Aufgabeband angetrieben werden. Ist die Hangabtriebskraft auf Grund des Massenstromes allerdings größer als der Bewegungswiderstand des Übergabesystems, so kann Energie vom Kettensystem über das Reibrad auf das Aufgabeband übertragen werden. Eine Energierückgewinnung ist demnach ebenfalls möglich.

**Figure 2:**
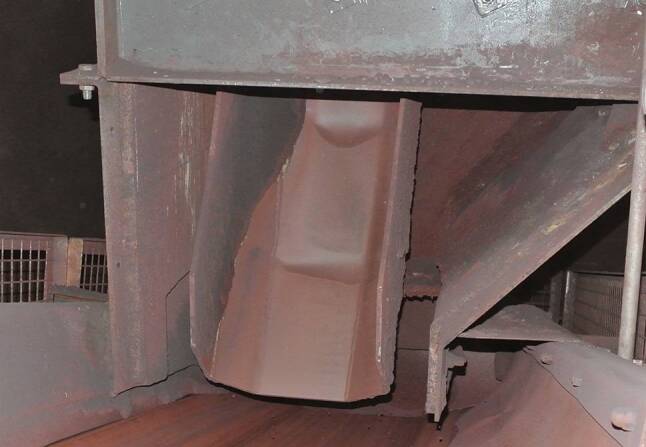

Da das dynamische Übergabesystem das Schüttgut unter einem bestimmten Winkel auf das Aufgabeband aufgibt, besitzt das Schüttgut eine Geschwindigkeitskomponente in Richtung der Förderrichtung des Aufgabebandes. Ein sogenannter „Soft Loading“ Effekt tritt auf. Durch diesen Effekt muss das Schüttgut um eine geringere Geschwindigkeitsdifferenz beschleunigt werden, wodurch weniger Leistung für den Betrieb des Aufgabebandes benötigt wird. Das System weist auch Verschleißvorteile gegenüber herkömmlichen Schurrensystemen auf. Durch die geringere Aufprallenergie des Schüttgutes beim Kontakt mit dem Aufgabeband und die gerichtete Aufgabe auf den Gurt, wird das Aufgabeband weniger Verschleiß ausgesetzt. Bei Standardschurren ist der Verschleiß immer auf bestimmte Bereiche konzentriert (siehe Abb. 2), durch das umlaufende Kettensystem ist dies nicht mehr der Fall, da sich die Kontaktzone auf die gesamte umlaufende Kette aufteilt.

**Figure 3:**
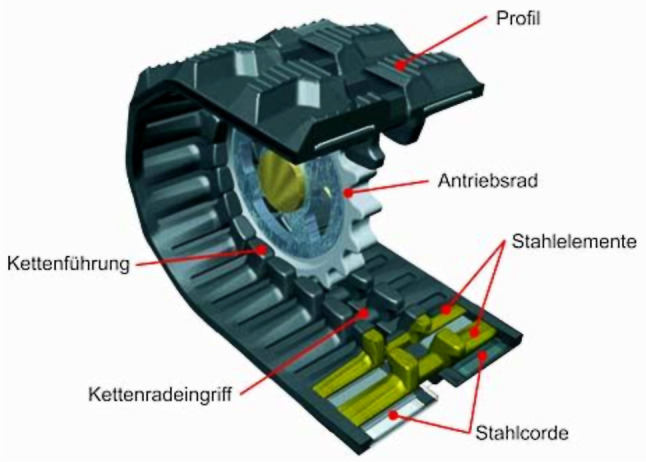

Durch die Reduktion der Fallhöhen bis zum Kontakt der Partikel mit dem Kettensystem bzw. dem Aufgabeband verringern sich die Kontaktgeschwindigkeiten der Partikel, wodurch die Schüttgutpartikel ebenfalls einem geringeren Verschleiß ausgesetzt sind. Unerwünschter Partikelbruch kann vermieden bzw. reduziert werden. Durch das umlaufende Kettensystem sind Verstopfungen der Übergabeschurre unwahrscheinlich. Bei sehr kohäsiven Schüttgütern ist es sinnvoll, unprofilierte Ketten zu verwenden, da diese mit zusätzlichen Gurtabstreifern zur Reinigung ausgerüstet werden können. Im Gegensatz zu Fördergurten weisen Gummiketten ein mittiges Führungsprofil auf (Abb. 3), in das die Kettenräder und Stützrollen eingreifen. Durch diese Führung kann es zu keinem Gurtschieflauf kommen. Ein derartiger Schieflauf entsteht beim Aufprall des Schüttgutes auf einen Gurt durch Aufprallkräfte, die in der Gurtebene z. B. 90° zur Förderrichtung wirken.

## Prototypentwicklung

**Figure 4:**
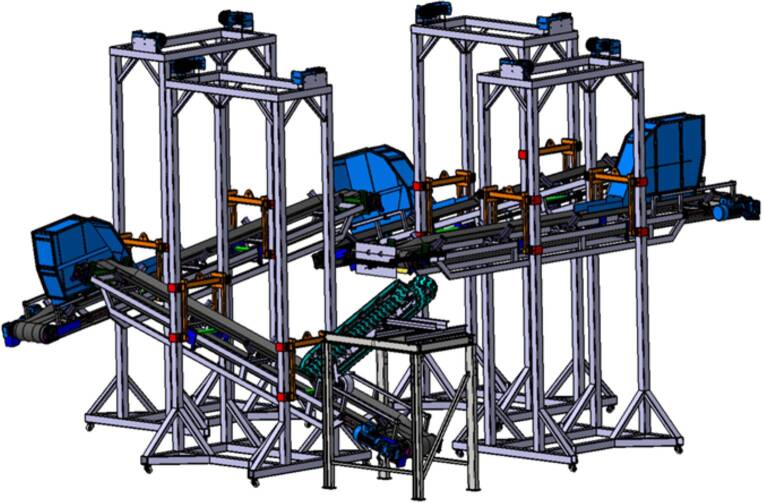

Um die Funktion des Systems zu bestätigen, ist ein Prototyp der Übergabeeinrichtung in den lehrstuhleigenen Förderkreislauf bestehend aus vier Gurtbandförderern, welche Schüttgüter im Kreis fördern können, an einer Übergabestelle eingebaut worden (siehe Abb. 4).

**Figure 5:**
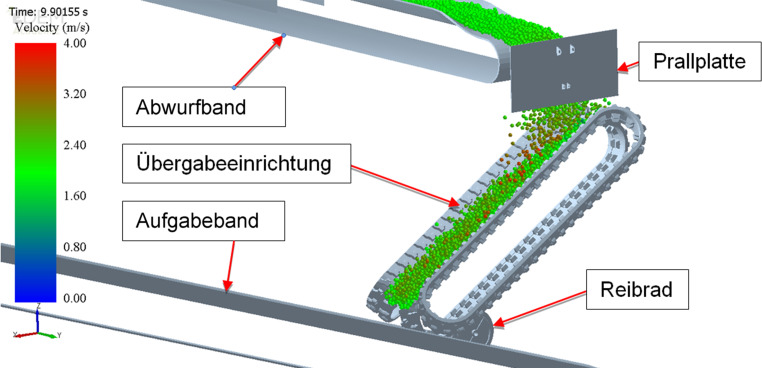

Zur Auslegung des Prototyps wurde unter anderem die „Diskrete Elemente Methode“ (DEM) eingesetzt (siehe Abb. 5). Dabei handelt es sich um eine Simulationsmethode, mit deren Hilfe die Bewegung einer großen Anzahl von Partikeln, wie es für Schüttgutbewegungen erforderlich ist, simuliert werden kann. Die Simulationen liefern Erkenntnisse bezüglich der Kräfte, die auf das System einwirken, der Leistung, der Partikelbelastung, der optimalen Geometrie und Position der Übergabeeinrichtung sowie der Verschleißsituation. Die Erkenntnisse aus den Simulationen fließen anschließend in die Dimensionierung des Systems ein.

Laut Simulation ergibt sich beispielsweise bei einem Massenstrom von 40 kg/s und 2 m/s Ketten- und Bandgeschwindigkeit, bei den gegebenen geometrischen Randbedingungen, eine Hangabtriebskraft von ca. 103 N. Die Hangabtriebskraft verursacht bei der gewählten Geschwindigkeit eine Leistung von ca. 206 W.

**Figure 6:**
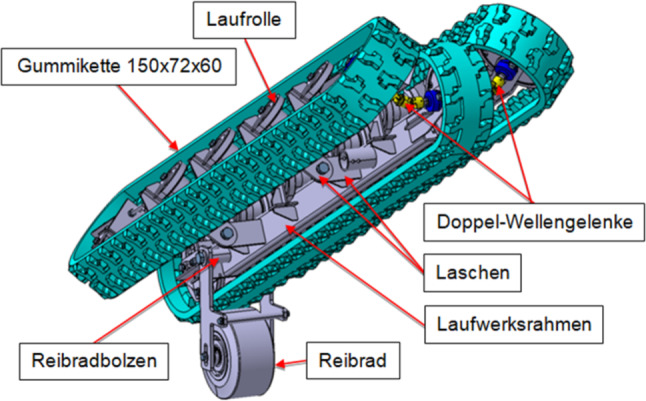

Nach Abschluss der Simulationsarbeiten konnte die exakte Position und die Abmessungen der Übergabeeinrichtung festgelegt werden, und es konnte mit der Ausdetaillierung der Konstruktion begonnen werden. Eine 3D-Darstellung des Kettensystems ist in Abb. 6 dargestellt.

Nach der Beendigung der Konstruktions- und Dimensionierungsarbeiten wurde der Prototyp am Lehrstuhl gefertigt und in den Förderkreislauf implementiert. Das Kettensystem ist aus drei 150 mm breiten Gummiketten mit einer Teilung von 72 mm und einem Achsabstand von 1740 mm aufgebaut. Die Ketten verfügen über je 60 Stahleinlagen und die Höhe eines „Kettenfahrwerks“ (Abstand Profilaußenfläche Last- zu Leertrum) beträgt 322 mm. Die Übergabehöhe zwischen Abwurf- und Aufgabeband beträgt ca. 1700 mm. Der Neigungswinkel der Ketten zur Horizontalen wurde mit ca. 34° eingestellt. Zur Übertragung der Antriebsleistung von Aufgabeband auf den mittleren Gummikettenstrang stehen drei verschiedene Reibradtypen zur Verfügung. Es handelt sich dabei um ein breites (Breite 130 mm, Durchmesser 250 mm) und ein schmales (Breite 50 mm, Durchmesser 250 mm) unprofiliertes Vollgummirad sowie um einen profilierten Luftreifen mit einer Breite von 85 mm und einem Durchmesser von 260 mm.

## Erkenntnisse der ersten Versuchsreihen

**Figure 7:**
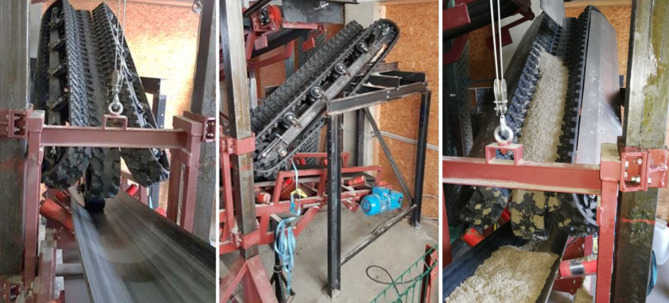

Bei der ersten Inbetriebnahme wurde das breite Vollgummireibrad verwendet. Es hat sich gezeigt, dass die Eigenmasse des Systems, welches sich teilweise am Reibrad abstützt, nicht ausreicht, um die Bewegung vom Gurt auf die Kette schlupffrei zu übertragen. Eine zusätzliche Anpresskraft musste mittels eines Spanngurtes aufgebracht werden. Voruntersuchungen bezüglich der Reibungsverhältnisse zwischen Rad und Gurt wurden mit dem schmalen Reibrad durchgeführt, da dieses ursprünglich verwendet werden sollte. Laut Messungen liegt ein Reibungskoeffizient von mindestens µ = 0,6 vor. Auf Grund der Eigenmasse und des ebenfalls gemessenen Bewegungswiderstandes des Kettensystems von ca. 803 N, müsste die für die Überwindung der Bewegungswiderstände nötige Leistung eigentlich übertragbar sein. Die für die Versuchseinrichtung in den benötigten Dimensionen verfügbaren Gummiketten weisen eine ungünstige Stollengeometrie auf, da der Mittelteil der Ketten perforiert ist und der Stollenabstand immer eine Vertiefung verursacht. Die mittlere Kette ist daher im mittleren Bereich mit einem Gummimaterial (Kaltvulkanisierung für Gurtreparaturen) verschlossen worden (siehe Abb. 7), um eine ebene durchgehende Kontaktfläche zum Reibrad zu erzeugen. Bei Gummiketten in größeren Dimensionen für den Industrieeinsatz ist dies nicht notwendig, da hier geeignete Kettengeometrien zur Verfügung stehen. Das verwendete Gummimaterial ist allerdings sehr weich, weshalb eine breitere Reifengeometrie verwendet werden musste. In Kombination mit dem breiten Vollgummirad reicht die Eigenmasse bzw. reichen die Reibungsverhältnisse nicht mehr aus, um die Bewegungswiderstände zu überwinden. Durch die Verwendung eines profilierten Luftreifens konnten die zusätzlich benötigten Anpresskräfte reduziert werden und die Leistung lässt sich problemlos übertragen. Die ursprüngliche Konstruktion der Anlage ist in weiterer Folge mit einem zusätzlichen Gestänge zur Erhöhung des Anpressdruckes ausgestattet worden.

**Figure 8:**
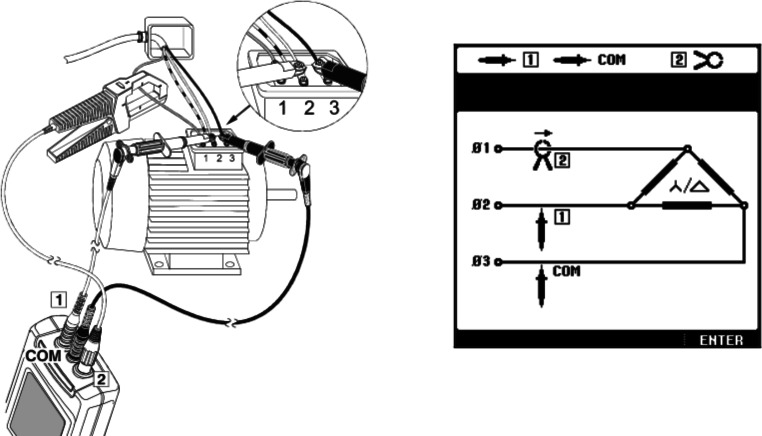

Zur Bestimmung der Leistungsfähigkeit und des realen Bewegungswiderstandes des Kettensystems wurden zahlreiche Leistungsmessungen unter den verschiedensten Randbedingungen durchgeführt. Exemplarisch werden im Nachfolgenden die Messergebnisse für einen Massenstrom von 13 kg/s (46,8 t/h) und einer Band- bzw. Kettengeschwindigkeit von 1,5 m/s vorgestellt. Zur Leistungsmessung wurde der Power Quality Analyzer 43B der Firma Fluke verwendet, wobei die Leistung direkt am Asynchronmotor (Nennleistung = 2,2 kW) des Aufgabebandes durchgeführt wurde. Das Messprinzip ist in Abb. 8 dargestellt.

**Figure 9:**
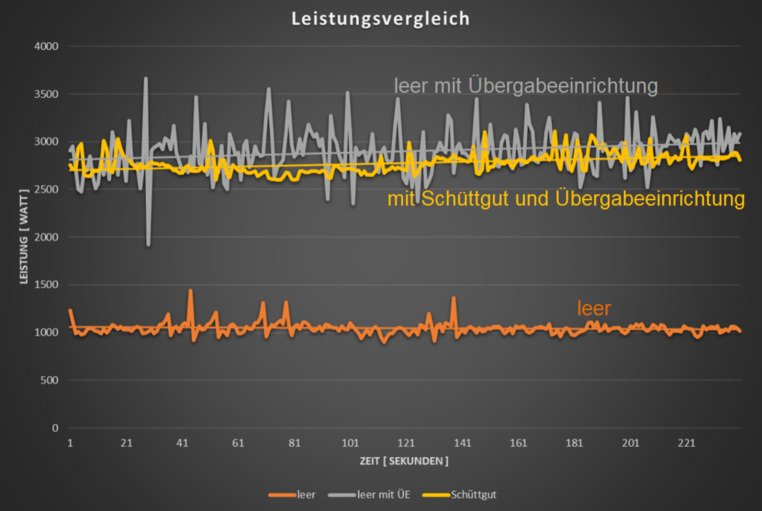

Das unbelastete Aufgabeband (ohne Schüttgut und ohne Übergabesystem) benötigt eine Antriebsleistung von 1045 W. Durch das Aufsetzen des Übergabesystems ohne Schüttguttransport erhöht sich die Antriebsleistung des Aufgabebandes auf 2898 W. Der Leistungsbedarf für das Kettensystem liegt demnach bei 1853 W. Wird nun das Gesamtsystem mit Schüttgut beaufschlagt, so reduziert sich die Leistungsaufnahme des Aufgabebandes auf 2775 W, was einer Reduktion um 123 W entspricht. Die Messkurven sind in (Abb. 9) ersichtlich. Der Leistungsbedarf des Aufgabebandes mit Schüttgut ohne Übergabesystem kann durch den derzeitigen Anlagenaufbau nicht bestimmt werden.

## Resümee und Aussicht

Die ersten Untersuchungen haben verdeutlicht, dass die Funktionalität des Systems gegeben ist. Die Leistungsübertragung durch ein Reibrad wurde realisiert, wobei sich ein profilierter luftgefüllter Reifen als besonders geeignet und kostengünstig erweist. Wie erwartet reduziert sich der Leistungsbedarf des Kettensystems durch die Aufgabe von Schüttgut. Der Massenstrom und die Bewegungsgeschwindigkeit im beladenen Zustand können derzeit nicht gesteigert werden, da der verwendete Antriebsmotor des Aufgabebandes unterdimensioniert ist. Der 2,2 kW Antrieb soll durch einen 4 kW Antrieb ersetzt werden, wodurch sich die Leistungsgrenze der Anlagenkonfiguration ermitteln lässt. Das Durchrieseln von trockenem, feinkörnigem Schüttgut mit sehr kleinem Schüttwinkel zwischen den einzelnen Kettensträngen, wird zukünftig durch die Verwendung geeigneter Kettenprofile mit entsprechender Kettenüberdeckung beseitigt. Für die Laborversuch konnten keine geeigneten Kettenprofile in den benötigten Dimensionen von Gummikettenlieferanten zugekauft werden. Zur Bestätigung der durch Simulationen ermittelten Verschleißvorteile ist ein Langzeitversuch an einer Industrieanlage geplant. Die Durchführung der Versuche an einer Industrieanlage unter Realbedingungen kann sich auf Grund der aktuellen COVID 19 Situation verzögern.
